# Evaluation of YouTube Videos as a Source of Information on Hepatosteatosis

**DOI:** 10.7759/cureus.46843

**Published:** 2023-10-11

**Authors:** Duygu Tutan, Muhammed Kaya

**Affiliations:** 1 Department of Internal Medicine, Erol Olçok Research and Training Hospital, Çorum, TUR; 2 Department of Gastroenterology, Hitit University Faculty of Medicine, Çorum, TUR

**Keywords:** internal medicine, video quality, youtube, gastroenterology, hepatosteatosis

## Abstract

Introduction

Individuals frequently turn to YouTube as a source of information about their medical conditions and potential treatment options. Among the common ailments affecting the general population, hepatosteatosis stands out due to its severe consequences in the absence of proper treatment. The primary objective of this study is to evaluate the quality of hepatosteatosis-related videos available on the YouTube platform, and the secondary objective is to determine if there is a difference in video quality between videos uploaded by medical professionals and other sources.

Methods

The process of selecting videos for this study involved evaluating their relevance after conducting a search using the keywords "hepatosteatosis," "fatty liver," and "hepatic steatosis" on YouTube. This search was conducted on August 18, 2023. From the search results, we identified and selected the top 50 most-watched videos in the English language. These selected videos were then rigorously assessed for their relevance and content by three independent medical professionals. Additionally, various descriptive attributes of each video, such as the upload date, subscriber count, view count, likes, dislikes, and comments, were meticulously recorded in the dataset. To determine the quality of these videos, we utilized three evaluation tools: the DISCERN Score, the Global Quality Score (GQS), and the Journal of the American Medical Association (JAMA) rating scales. We have used the median±interquartile range (IQR), mean±standard deviation (SD), and the range of minimum to maximum values to convey descriptive statistics. The distribution was evaluated with the Shapiro-Wilks test. Spearman correlation analysis was used to identify relationships between variables. The association between quality indicators and data was examined using multiple regression analysis. The Mann-Whitney U test was used to determine significant differences between groups. A statistical significance level of 0.05 was considered significant.

Results

Our study revealed notable statistical differences in DISCERN scores when comparing videos uploaded by medical doctors to those uploaded by individuals without medical qualifications (p < 0.001). Likewise, in the comparisons between these two groups, videos created by healthcare professionals consistently demonstrated significantly higher quality scores in both the JAMA and GQS evaluations (p < 0.001 for both comparisons). This suggests that videos uploaded by medical professionals tend to provide higher-quality information on the topic of hepatosteatosis compared to those uploaded by non-medical individuals. Video length and comment counts were also found to be significant in the multivariate linear regression analysis and were predictive of the DISCERN score (p = 0.047 and p = 0.037, respectively).

Conclusions

The quality of information related to hepatosteatosis on YouTube varies significantly. Surprisingly, there is no noticeable difference in terms of views and popularity between helpful and potentially misleading videos. For individuals seeking reliable information, it is advisable to prioritize videos uploaded by medical professionals. Paying attention to the qualifications of the content creator rather than the video's popularity or view count is crucial when seeking accurate and trustworthy information on hepatosteatosis.

## Introduction

YouTube, an online video-sharing platform, has amassed a significant worldwide viewership, reaching billions of users on a daily basis. This notable increase in user participation and viewership can be attributed to the enhanced accessibility offered by social media platforms and the continual evolution of Internet infrastructure [1]. Multiple studies have provided evidence that both patients and healthcare professionals utilize videos disseminated on the YouTube platform as a valuable source of informative content [2,3].

In recent times, a new business model has emerged on the YouTube platform, involving the upload of videos with the intention of generating revenue based on the number of views. As a result, there has been a notable shift towards uploading videos with the primary goal of increasing click-through rates and view counts, sometimes at the expense of the fundamental authenticity of the content [4]. This phenomenon has prompted a reflection on the integrity of video content quality, with a particular emphasis on health-related videos. Scholarly inquiry has highlighted the importance of YouTube as a potential resource for young individuals seeking health-related information. Empirical studies have demonstrated that content delivered by healthcare professionals has the potential to support learning and improve knowledge acquisition [3].

Both healthcare professionals and patients actively utilize social media platforms as sources of information. Given the substantial volume of videos covering a wide range of topics, numerous research studies in the healthcare domain have been dedicated to assessing the quality, relevance, and usefulness of videos hosted on YouTube [5-7].

Hepatosteatosis, commonly referred to as fatty liver disease, is a medical condition characterized by the abnormal buildup of lipids, particularly triglycerides, within the liver [8,9]. This condition is a prevalent metabolic disorder with various contributing factors, including excessive consumption of fructose, dysbiosis (imbalanced gut microbiota), inflammation, and genetic predisposition [9-11].

The consumption of fructose has been found to stimulate a process known as de novo lipogenesis (DNL), which involves the creation of new fatty acids from non-lipid sources. Notably, fructose is a more potent inducer of hepatic DNL compared to glucose [10]. Excessive fructose intake can have detrimental effects, including disruption of the gut microbiota balance, known as dysbiosis, and the downregulation of tight junction proteins. These changes can lead to the deterioration of the intestinal barrier and result in low-grade endotoxemia, where bacterial toxins enter the bloodstream [10]. These interconnected factors can contribute to the development of hepatosteatosis, or fatty liver disease.

Inflammation represents another significant factor that can exacerbate the process of fructose-stimulated de novo lipogenesis and contribute to hepatosteatosis. Inflammatory pathways have the potential to disrupt lipid metabolism, facilitating the buildup of triglycerides in the liver. Specific inflammatory signaling pathways, such as those mediated by toll-like receptors and macrophage activation, have been identified as contributors to the development of hepatosteatosis [10]. Additionally, genetic factors have been implicated in the onset of hepatosteatosis. Mutations in genes responsible for lipid metabolism and homeostasis, such as PNPLA3 and KBTBD2, have been linked to this condition [12,13]. These genetic variations can influence the processing and storage of lipids within the liver, ultimately resulting in triglyceride accumulation [12,13].

Hepatosteatosis can progress into more severe forms of liver disease, notably non-alcoholic fatty liver disease (NAFLD), characterized by liver inflammation and damage to liver cells [10]. NAFLD has the potential to advance into liver fibrosis, cirrhosis, and even hepatocellular carcinoma (HCC) [10,11]. In the United States, NAFLD affects an estimated 30% of adults and stands as a major cause of liver failure and liver cancer [8]. The worldwide prevalence of hepatosteatosis is significant and continues to increase; it is estimated that NAFLD affects approximately 25% of the global population [14]. This condition is closely linked to metabolic syndrome and exhibits strong associations with obesity and diabetes [15,16]. Treatment strategies for hepatosteatosis predominantly revolve around lifestyle modifications, including dietary adjustments and increased physical activity, aimed at reducing liver fat accumulation [17,18]. In some instances, pharmacological interventions may be necessary to effectively manage the condition [18].

Precise diagnostic assessments and well-considered treatment approaches are essential for achieving the best possible outcomes for patients. In the case of individuals dealing with hepatosteatosis, seeking early guidance from healthcare experts is crucial. This can lead to the development of personalized management plans that have the potential to improve health and overall quality of life. Given the prevalence of YouTube as a primary source of information in today's world, it often serves as one of the initial resources patients turn to when seeking information about their medical conditions.

The primary aim of this study is to conduct an impartial evaluation of the quality of videos related to hepatosteatosis. This inquiry was initiated based on the notion that the abundant availability of videos elucidating hepatosteatosis has the potential to contribute to the well-being of patients by providing informative insights and guidance concerning their medical condition.

## Materials and methods

On August 18, 2023, our search on the YouTube platform involved the use of the keywords "hepatosteatosis," "fatty liver," and "hepatic steatosis." We then sorted the search results according to the most-viewed videos. Subsequently, we applied our exclusion criteria, removing videos with redundant content, those presented in languages other than English, videos lacking relevance to hepatosteatosis, and videos with a duration shorter than one minute. This selection process resulted in the compilation of the 50 most watched videos that met the stringent criteria and were deemed highly relevant for the study. During this process, 48 videos were excluded due to their short duration or being non-English-language videos.

All 50 selected videos underwent a thorough evaluation conducted independently by three different medical practitioners (one internal medicine specialist, one gastroenterologist, and one general surgeon). This assessment primarily aimed to evaluate the relevance, content, and overall quality of each video. Additionally, the dataset was augmented with descriptive attributes associated with each video. These attributes included details such as the upload date, the identity of the video uploader, the uploader's subscriber count, the number of views, the degree of appreciation indicated by likes, the level of disapproval marked by dislikes, and the comments provided by viewers below the video content.

The evaluation of video quality was carried out utilizing three distinct rating scales: DISCERN, Global Quality Score (GQS), and the Journal of the American Medical Association (JAMA) Framework. The DISCERN scoring system, renowned for its comprehensiveness, is structured into two distinct clusters, incorporating a total of 16 questions. These questions collectively serve as a comprehensive set of criteria for evaluation [19]. Under this assessment framework, the initial section of the evaluation pertains to safety considerations, while the subsequent section is dedicated to appraising the quality of information concerning available treatment options. It is worth noting that the grading for the sixteenth question is conducted independently of the ratings given for the preceding 15 questions. Consequently, the point scale delineates quality levels as follows: Scores ranging from 16 to 26 indicate an extremely low-quality standard, while scores falling within the range of 27 to 38 characterize videos of low quality. Videos accumulating points within the range of 39 to 50 signify a medium level of quality, whereas those achieving scores from 51 to 62 indicate an acceptable quality standard. Notably, videos accumulating points spanning from 63 to 75 exemplify an exceptional quality benchmark [20,21].

The comprehensive evaluation of the entire collection of video content was carried out using the Global Quality Scale (GQS), which employs a 5-point scale. This evaluative framework considers multiple dimensions, including the accessibility of the conveyed information, the quality of the information provided, the coherence and continuity in the presentation of information, and an assessment of the practical usefulness perceived by the reviewer in terms of its potential relevance to patients [22].

Furthermore, the data underwent evaluation using the JAMA scoring system. This system assesses video quality by examining several crucial aspects, including authorship, attribution, description, and validity. Each of these criteria is assigned a value of either 0 or 1, reflecting the degree to which the specified criteria are met. It is important to note that within the context of the JAMA evaluation, a score of 1 point corresponds to insufficient information, while a score of 2 to 3 points indicates partially sufficient information. Remarkably, a score of 4 points signifies the attainment of information of notable quality [23].

The evaluation of video popularity was accomplished by employing the Video Power Index (VPI), a metric computed as the product of the number of likes multiplied by 100, divided by the sum of likes and dislikes. Additionally, to address potential bias arising from the time factor, the daily view count, which is calculated as the total views divided by the days elapsed since upload, was utilized. This methodology aims to account for the possibility that a video might accumulate more views simply because it was uploaded earlier on the YouTube platform [24,25].

The videos were methodically classified into two distinct groups based on the differentiation between content producers possessing medical expertise (such as medical doctors or healthcare institutions) and those lacking such expertise (including personal YouTube channels or non-medical entities). Furthermore, video durations falling below and exceeding the six-minute threshold, as well as release dates preceding three years (indicating new videos) and extending beyond three years (representing older videos), were subjected to stratification. Additionally, video engagement was assessed by stratifying the daily view count as either below or above 1750. The VPI was similarly divided into segments lower and higher than 98. Moreover, video comment frequency per year was categorized as either above or below 500. These distinct subgroups were systematically examined to provide a nuanced perspective on video quality and relevance within each respective category. The evaluation encompassed an analysis of video quality coupled with an exploration of the interrelationships between the defined subgroups.

In March 2021, YouTube introduced a change in which the count of dislikes for videos was hidden from view. This alteration presented a challenge when calculating the VPI score, a crucial metric for our study. To overcome this challenge, we employed the "Return YouTube Dislikes" Chrome extension to access the necessary dislike count information.

Formal approval from an institutional ethics review board was not necessary for the execution of this study, and no external funding was obtained for this research.

Statistical analyses were performed utilizing the IBM SPSS Statistics for Windows software (version 26; IBM Corp., Armonk, NY, USA). To provide a comprehensive characterization of the data, we computed descriptive statistics, including measures such as median±interquartile range (IQR), mean±standard deviation (SD), and the range of minimum to maximum values. The conformity of each dataset within its respective group to a normal distribution was evaluated using the Shapiro-Wilk test. Relationships between variables were identified through Spearman correlation analysis. The association between quality indicators and the data was examined using multiple regression analysis. To determine significant differences between groups, the Mann-Whitney U test was used. A statistical significance level of 0.05 was deemed significant.

## Results

Upon evaluating the top 50 most-viewed videos, a collective total of 110,840,472 views was documented. These videos exhibited an average duration of 545±336 seconds, with a range spanning from a minimum of 82 seconds to a maximum of 2041 seconds. It is noteworthy that the most widely watched video garnered an impressive 11,786,302 views. In terms of daily viewership, the highest observed count reached 19,710 views per day, while the average daily viewership stood at 3,864 views. Further statistical details are in Table 1. The videos exhibited an average VPI value of 97.51±2.08, a mean DISCERN score of 50±18, a mean JAMA rating of 2±1, and a mean GQS value of 2±1.

**Table 1:** Data of 50 most watched hepatosteatosis videos on the YouTube platform SD: standard deviation, GQS: Global Quality Score, JAMA: Journal of the American Medical Association, VPI: Video Popularity Index, IQR: interquartile range

Variables	Mean±SD	Median±IQR	Minimum-maximum
View count	2216809±2682682	1274348±1645583	524752-11786302
Days from upload	1009±782	825±1261	107-3113
Daily view count	3864±4786	1815±4203	201-19710
Video length (seconds)	545±336	487±408	82-2041
Comment count	2139±2662	1295±1645	65-11274
Comment per Year	1307±1820	583±1219	26-6912
Like count	45826±56540	26500±38000	4500-255000
Dislike count	1154±2213	547±917	0-14000
VPI	97.51±2.08	98.12±1.9	90.91-100
DISCERN	50±18	46±33	27-78
GQS	2±1	2±2	0-4
JAMA	2±1	2±2	0-4

**Figure 1:**
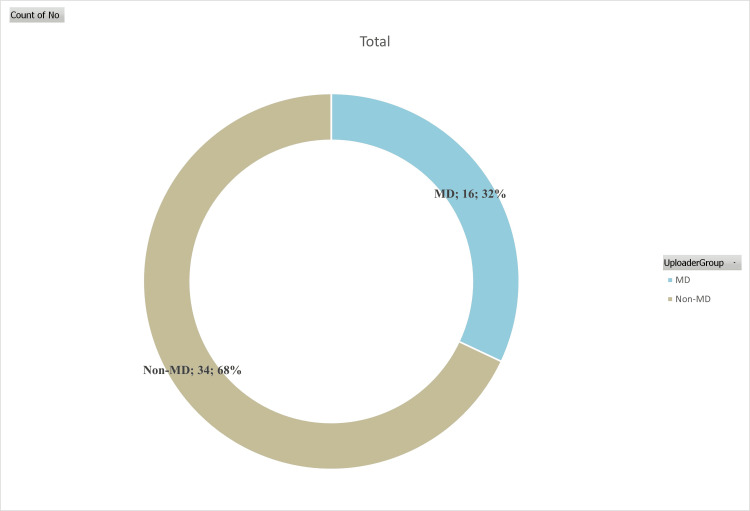
Pie chart of videos according to uploader

**Figure 2:**
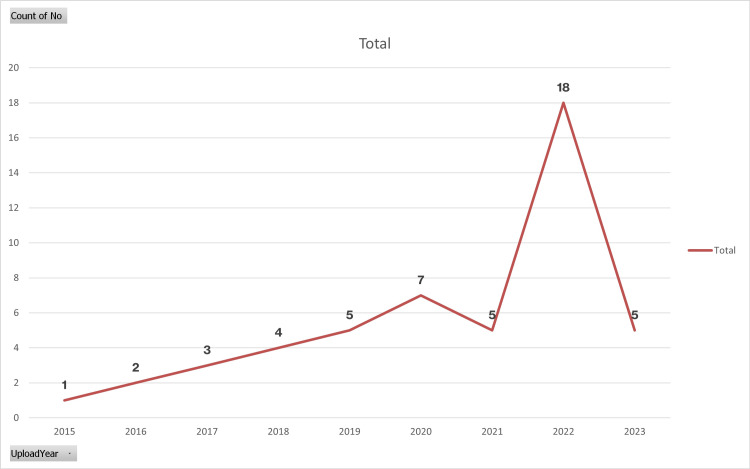
Line graph of videos about hepatosteatosis according to upload years

Of the individuals who contributed content to the YouTube platform, 16 (32%) were identified as healthcare professionals, while the remaining 34 (68%) did not hold such professional credentials (Figure 1 and Table 2). The upload dates of the videos ranged from 2015 to 2023 (Figure 2).

Based on the DISCERN score, it was ascertained that 17 videos attained an exceptional quality rating, with an additional two videos being classified as possessing acceptable quality. An additional 13 videos were evaluated as having medium quality, while 18 videos were categorized as demonstrating poor quality. Notably, among the videos created by healthcare professionals, none were assessed as being of poor quality. Conversely, only two videos of exceptional quality were produced by non-professional content creators.

A statistically significant difference was identified in relation to DISCERN scores between videos uploaded by medical professionals and those uploaded by non-physician individuals (p < 0.001). Similarly, when comparing these two distinct groups, it was found that videos produced by medical professionals exhibited significantly higher quality scores in both JAMA and GQS evaluations (p < 0.001 for both comparisons). There were no significant differences observed in terms of time from upload, subscriber count, view count, daily view count, like count, dislike count, VPI, or video length between videos uploaded by professionals and videos uploaded by non-physician individuals (p = 0.917, p = 0.068, p = 0.647, p = 0.724, p = 0.603, p = 0.724, p = 0.328, and p = 0.308, respectively).

No notable distinction was observed in terms of quality scores when considering videos uploaded within the last three years versus those exceeding this time threshold (Table 2). Similarly, when segregating videos based on daily view counts below or above 1750 and a video length shorter than six minutes or longer, no substantial variance was observed in DISCERN, GQS, and JAMA scores between the respective groups (Table 2). No discernible difference in quality was evident when contrasting videos based on VPI scores below and above 98 (Table 2). Notably, videos with annual comment counts higher than 500 exhibited higher DISCERN and JAMA scores compared to their lower counterparts, although the GQS scores demonstrated no significant differences (p = 0.013, p = 0.004, and p = 0.066, respectively).

**Table 2:** Relationship between categoric variables and video quality scores GQS: Global quality score, JAMA: Journal of the American Medical Association, VPI: Video Popularity Index, IQR: interquartile range, MD: medical doctor

Variables		Count (%)	DISCERN	Statistical significance	GQS	Statistical significance	JAMA	Statistical significance
			Median±IQR		Median±IQR		Median±IQR	
Professionality	Non-professional	34 (68%)	38±14	<0.001	2±1	<0.001	1±1	<0.001
	Professional (MD)	16 (32%)	72±8		3±1		3±2	
Upload date	Old (>3 years)	22 (44%)	45±31	0.822	2±2	0.367	2±2	0.524
	New (<3 years)	28 (56%)	46±36		3±1		2±2	
Daily view count	Daily view count < 1750	24 (48%)	40±34	0.150	2±2	0.004	1±2	0.135
	Daily view count > 1750	26 (52%)	49±33		3±2		2±2	
Video duration	Video length < 6 mins	17 (34%)	39±10	0.255	2±1	0.074	1±1	0.096
	Video Length >6 mins	33 (66%)	50±36		3±1		2±2	
Comment/year	Comment/year <500	23 (46%)	39±18	0.013	2±2	0.066	1±1	0.004
	Comment/year > 500	27 (54%)	54±34		3±2		3±1	
VPI	VPI < 98	21 (42%)	42±31	0.969	2±1	0.628	2±2	0.823
	VPI > 98	29 (58%)	47±32		2±2		2±2	

The results of our study revealed a statistically significant positive correlation between the quality scores, with p-values below 0.001 for all measurements, as detailed in Table 3. A significant difference in terms of uploader professionalism was identified between videos categorized as useful (DISCERN > 50) and not useful (DISCERN < 50) (p < 0.001). Notably, all videos uploaded by professionals fell into the useful group. Conversely, no significant differences were observed in relation to the time elapsed since upload, subscriber count, view count, daily view count, or VPI (Table 4). However, significant differences were evident with regard to video length, comment count, comment per year, like count, and dislike count (Table 4). A subsequent multivariate linear regression analysis was conducted to identify independent predictors. In the context of multivariate linear regression analysis, it was determined that variables such as VPI, view count, likes, dislikes, subscriber counts, and upload dates did not have a significant impact on DISCERN scores (Table 5). However, factors that did significantly influence the scores included the professional status of the content uploaders, video duration, and comment counts (p < 0.001, p = 0.047, and p = 0.037, respectively).

**Table 3:** Correlation between quality scores GQS: Global quality score, JAMA: Journal of the American Medical Association, r: correlation coefficient, p: statistically significant

Variables		DISCERN	GQS	JAMA
DISCERN	r	1		
	p			
GQS	r	0.872	1	
	p	<0.001		
JAMA	r	0.685	0.677	1
	p	<0.001	<0.001	

**Table 4:** Comparison of variables between useful and not useful videos according to DISCERN GQS: Global quality score, JAMA: Journal of the American Medical Association, VPI: Video Popularity Index

Variables		Not Useful	Useful	Statistical significance
		DISCERN < 50	DISCERN > 50	
Uploader professionality	Non-professional	30 (100%)	4 (20%)	<0.001
	Professional	0 (0%)	16 (80%)	
Views		1,068,851±1,096,969	1,699,153±1,970,993	0.185
Subscribers		3,040,000±10,034,000	2,770,000±8,350,500	0.540
Days from upload		825±909	740±1344	0.874
Daily view count		1719±3926	1921±5451	0.205
Video Length		384±352	547±345	0.045
Comment count		879±974	1963±3544	0.005
Comment/year		307±618	1052±2083	0.005
Like count		20000±19000	44500±84,500	0.025
Dislike count		461±412	733±1515	0.034
VPI		98.21±1.86	98.02±2.12	0.797
DISCERN		38±13	71±9	<0.001
GQS		2±1	3±1	<0.001
JAMA		1±1	3±1	<0.001

**Table 5:** Results of multivariate linear regression analysis of DISCERN score and variables VPI: Video Popularity Index, Nagelkerke R square = 0.827, significance of the model is <0.001

Coefficients	B (%95 confidence interval)	Statistical significance
(Constant)	126.726 (−42.418 to 295.871)	0.138
Subscribers	2.18E-07 (0.000–0.000)	0.623
Views	4.71E-06 (0.000–0.000)	0.099
Days from upload	0 (−0.005 to 0.004)	0.863
Daily view count	−0.002 (−0.005 to 0.000)	0.085
Video Length	0.009 (0.000–0.019)	0.047
Comment count	−0.005 (−0.010 to 0.000)	0.037
Comment/year	0.004 (−0.001 to 0.009)	0.132
Likes	0 (0.000–0.000)	0.175
Dislikes	0 (−0.002 to 0.003)	0.736
VPI	−0.985 (−2.754 to 0.784)	0.267
Uploader professionality	34.172 (27.807–40.536)	<0.001

## Discussion

The complex YouTube algorithm, designed to navigate and categorize the immense pool of videos available on the platform to identify those of superior quality and pertinence, functions through an intricate and ever-evolving process heavily shaped by the feedback and interactions of viewers [26]. It is important to acknowledge that search results for hepatosteatosis may vary when conducted by different users at different times. Nevertheless, our study sought to mitigate this variability by focusing on evaluating the top 50 most-watched videos pertaining to hepatosteatosis.

The prevalence of hepatosteatosis is notably high, with estimations suggesting its impact on up to 1 billion individuals worldwide [14]. This condition is particularly prevalent among those who are obese and diabetic [15]. The growing prevalence of NAFLD is a concerning trend, given its potential to advance into more severe liver diseases like fibrosis, cirrhosis, and hepatocellular carcinoma [16]. The increasing global burden of hepatosteatosis underscores the urgency for effective prevention and management strategies. Rapid access to quality information can empower patients with a better understanding of the disease process, facilitate early professional intervention, and safeguard them from the associated deterioration in their quality of life.

In our research, we identified that videos uploaded by medical professionals achieved higher JAMA, GQS, and DISCERN scores. This highlights the possibility that YouTube can serve as a source of accurate and reliable information about hepatosteatosis, especially when the content is created by knowledgeable experts in the field. On the contrary, the presence of low-quality information on YouTube poses the risk of misleading patients and potentially leading to incorrect decisions. Moreover, this misinformation could complicate the patient-physician relationship.

It is important to note that factors other than quality indicators, such as view counts, likes/dislikes, and related metrics, did not show a significant correlation with video quality in our study. This emphasizes the importance of considering the qualifications of the content creator rather than relying solely on popularity metrics when seeking reliable medical information on YouTube.

The issue of information pollution, which is pervasive across the internet, is also evident on the YouTube platform. Our study revealed that 32% of the videos were uploaded by healthcare professionals and generally demonstrated high-quality content. However, the remaining 68% of videos were contributed by individuals or entities lacking medical expertise. This diversity in content creators, combined with a lack of regulatory oversight, contributes to the information pollution on YouTube. These findings are consistent with previous research, including the work of Turhan et al. [3], which also highlighted these concerns. Our study aligns with the existing literature, emphasizing that videos produced by non-physicians tend to exhibit lower-quality content [6,27].

YouTube has evolved into a substantial information source for both patients and healthcare practitioners. However, the platform's algorithm-driven search results primarily prioritize metrics like views and comments, often at the expense of content quality. This emphasizes the importance of considering the credibility and expertise of content creators when seeking reliable information on medical topics [28]. The heterogeneous nature of YouTube upload sources hinders the establishment of a standardized benchmark for video quality. Additionally, videos uploaded earlier tend to accrue more views over time. To address this time bias, daily view counts were calculated as a corrective measure in our study.

It is worth noting that many previous studies have consistently shown that videos uploaded by medical professionals generally have higher quality compared to those produced by non-physicians. However, an interesting observation is that physician-uploaded videos may sometimes receive fewer views. This is an important aspect to consider in the realm of online health information [29]. In our study, we did not find any statistically significant differences in view counts between videos uploaded by physicians and those uploaded by non-physicians, which is different from some previous observations. Additionally, there were no significant differences in terms of interaction when considering Video Popularity Index (VPI) rates. It is worth mentioning that while there have been reports of poor-quality videos gaining more popularity than higher-quality ones, our study did not support such findings in the context of hepatosteatosis [30].

A major limitation of our study is the absence of a universally accepted gold standard for evaluating the quality of YouTube videos. While the JAMA, DISCERN, and GQS metrics were not originally designed for assessing YouTube video quality, they have been commonly used in many studies, including ours, due to the lack of a more specialized tool for this purpose [3,6]. These evaluation systems have been widely recognized for their utility in assessing video quality [3,5,6]. Our study aimed to bolster the validation process by involving three separate medical professionals as reviewers. Nevertheless, it is conceivable that a more extensive panel of reviewers could further enhance the validation process. Additionally, the ever-evolving nature of the YouTube platform, marked by the daily addition of millions of new videos, raises the possibility that our evaluations may pertain specifically to the reviewed time frame. Another potential limitation relates to the relatively modest number of videos included in the study. However, the substantial total view count of 110,840,472 underscores the videos' impact and thus the study's significance.

## Conclusions

YouTube has become a widely visited platform for both patients and healthcare professionals. While the range of video quality on this platform is extensive, it is important to note that lower-quality videos can achieve similar viewership as higher-quality ones. The landscape of information related to hepatosteatosis on YouTube exhibits significant variability in terms of quality. Interestingly, there is no apparent difference in terms of views and popularity between informative videos and potentially misleading ones. For both patients and medical practitioners, selecting videos uploaded by healthcare professionals is a more advisable choice as an information source. Emphasizing the identity of the content uploader over factors like video popularity or view count is crucial.

## Appendices

**Table 6:** DISCERN scoring system

Section	Questions	No	Partly			Yes
Reliability	1. Explicit aims	1	2	3	4	5
	2. Aims achieved	1	2	3	4	5
	3. Relevance to patients	1	2	3	4	5
	4. Source of information	1	2	3	4	5
	5. Currency (date) of information	1	2	3	4	5
	6. Bias and balance	1	2	3	4	5
	7. Additional sources of information	1	2	3	4	5
	8. Reference to areas of uncertainty	1	2	3	4	5
Quality	9. How treatment works	1	2	3	4	5
	10. Benefits of treatment	1	2	3	4	5
	11. Risks of treatment	1	2	3	4	5
	12. No treatment options	1	2	3	4	5
	13. Quality of life	1	2	3	4	5
	14. Other treatment options	1	2	3	4	5
	15. Shared decision making	1	2	3	4	5
	16. The overall quality of the publication as a source of information	1	2	3	4	5

**Table 7:** Global Quality Scale criteria

Score	Global score description
1	Poor quality, poor flow of the site, most information missing, not at all useful for patients
2	Generally poor quality and poor flow, some information listed but many important topics missing, of very limited use to patients
3	Moderate quality, suboptimal flow, some important information is adequately discussed but others poorly discussed, somewhat useful for patients
4	Good quality and generally good flow, most of the relevant information is listed, but some topics not covered, useful for patients
5	Excellent quality and excellent flow, very useful for patients

**Table 8:** Journal of the American Medical Association scoring system

Subsection	Explanation
Authorship	Authors and contributors, their affiliations, and relevant credentials should be provided
Attribution	References and sources for all content should be listed clearly, and all relevant copyright information should be noted
Disclosure	Ownership should be prominently and fully disclosed, as should any sponsorship, advertising, underwriting, commercial funding arrangements or support, or potential conflicts of interest
Currency	Dates when content was posted and updated should be indicated

## References

[1] Ng FK, Wallace S, Coe B (2020). From smartphone to bed-side: exploring the use of social media to disseminate recommendations from the National Tracheostomy Safety Project to front-line clinical staff. Anaesthesia.

[2] Madathil KC, Rivera-Rodriguez AJ, Greenstein JS, Gramopadhye AK (2015). Healthcare information on YouTube: a systematic review. Health Informatics J.

[3] Turhan S, Akdağlı Ekici A (2023). YouTube as an information source of transversus abdominis plane block. Turk J Clin Lab.

[4] Varshney D, Vishwakarma DK (2021). A unified approach for detection of Clickbait videos on YouTube using cognitive evidences. Appl Intell (Dordr).

[5] Turhan VB, Ünsal A (2021). Evaluation of the quality of videos on hemorrhoidal disease on YouTube™. Turk J Colorectal Dis.

[6] Erdogan Kaya A, Erdogan Akturk B (2023). Quality and content analysis: can YouTube videos on agoraphobia be considered a reliable source?. Cureus.

[7] So H, Kim DW, Hwang JS, Ko SW (2022). YouTube as a source of information on endoscopic retrograde cholangiopancreatography. Medicine (Baltimore).

[8] Parker BL, Calkin AC, Seldin MM (2019). An integrative systems genetic analysis of mammalian lipid metabolism. Nature.

[9] Du C, Yang W, Yu Z (2022). Rheb promotes triglyceride secretion and ameliorates diet-induced steatosis in the liver. Front Cell Dev Biol.

[10] Todoric J, Di Caro G, Reibe S (2020). Fructose stimulated de novo lipogenesis is promoted by inflammation. Nat Metab.

[11] Lawan A, Zhang L, Gatzke F (2015). Hepatic mitogen-activated protein kinase phosphatase 1 selectively regulates glucose metabolism and energy homeostasis. Mol Cell Biol.

[12] Zhang Z, Gallagher T, Scherer PE, Beutler B (2020). Tissue-specific disruption of Kbtbd2 uncovers adipocyte-intrinsic and -extrinsic features of the teeny lipodystrophy syndrome. Proc Natl Acad Sci U S A.

[13] Sun Z, Lazar MA (2013). Dissociating fatty liver and diabetes. Trends Endocrinol Metab.

[14] Eskin F, Şenel E (2022). Holistic analysis of hepatosteatosis literature: a scientometric study of global hepatosteatosis publications between 1980 and 2019. J Med Palliat Care.

[15] Yoshino S, Iwasaki Y, Matsumoto S (2020). Administration of small-molecule guanabenz acetate attenuates fatty liver and hyperglycemia associated with obesity. Sci Rep.

[16] Isaza SC, Del Pozo-Maroto E, Domínguez-Alcón L, Elbouayadi L, González-Rodríguez Á, García-Monzón C (2020). Hypoxia and non-alcoholic fatty liver disease. Front Med (Lausanne).

[17] Jump DB, Depner CM, Tripathy S, Lytle KA (2015). Potential for dietary ω-3 fatty acids to prevent nonalcoholic fatty liver disease and reduce the risk of primary liver cancer. Adv Nutr.

[18] Mahzari A, Li S, Zhou X (2019). Matrine protects against MCD-induced development of NASH via upregulating Hsp72 and downregulating mTOR in a manner distinctive from metformin. Front Pharmacol.

[19] Keskinkılıç Yağız B, Yalaza M, Sapmaz A (2021). Is Youtube a potential training source for total extraperitoneal laparoscopic inguinal hernia repair?. Surg Endosc.

[20] Kaicker J, Borg Debono V, Dang W, Buckley N, Thabane L (2010). Assessment of the quality and variability of health information on chronic pain websites using the DISCERN instrument. BMC Med.

[21] Charnock D, Shepperd S, Needham G, Gann R (1999). DISCERN: an instrument for judging the quality of written consumer health information on treatment choices. J Epidemiol Community Health.

[22] Langille M, Bernard A, Rodgers C, Hughes S, Leddin D, van Zanten SV (2010). Systematic review of the quality of patient information on the internet regarding inflammatory bowel disease treatments. Clin Gastroenterol Hepatol.

[23] Batar N, Kermen S, Sevdin S, Yıldız N, Güçlü D (2020). Assessment of the quality and reliability of information on nutrition after bariatric surgery on YouTube. Obes Surg.

[24] Erdem MN, Karaca S (2018). Evaluating the accuracy and quality of the information in kyphosis videos shared on YouTube. Spine (Phila Pa 1976).

[25] Celik H, Polat O, Ozcan C, Camur S, Kilinc BE, Uzun M (2020). Assessment of the quality and reliability of the information on rotator cuff repair on YouTube. Orthop Traumatol Surg Res.

[26] Fyfield M, Henderson M, Phillips M (2021). Navigating four billion videos: teacher search strategies and the YouTube algorithm. Learn Media Technol.

[27] Kumar N, Pandey A, Venkatraman A, Garg N (2014). Are video sharing web sites a useful source of information on hypertension?. J Am Soc Hypertens.

[28] Lobato R (2016). The cultural logic of digital intermediaries: YouTube multichannel networks. Convergence.

[29] Desai T, Shariff A, Dhingra V, Minhas D, Eure M, Kats M (2013). Is content really king? An objective analysis of the public's response to medical videos on YouTube. PLoS One.

[30] Tartaglione JP, Rosenbaum AJ, Abousayed M, Hushmendy SF, DiPreta JA (2016). Evaluating the quality, accuracy, and readability of online resources pertaining to hallux valgus. Foot Ankle Spec.

